# SEM Evaluation of Thermal Effects Produced by a 445 nm Laser on Implant Surfaces

**DOI:** 10.3390/dj11060148

**Published:** 2023-06-07

**Authors:** Daniele Pergolini, Gaspare Palaia, Riccardo De Angelis, Federica Rocchetti, Gian Marco Podda, Gianluca Tenore, Alessandro Del Vecchio, Michela Relucenti, Umberto Romeo

**Affiliations:** 1 Department of Oral Sciences and Maxillofacial Surgery, Sapienza University of Rome, Via Caserta 6, 00161 Rome, Italy; 2 Department of Anatomical, Histological, Forensic and Orthopedic Sciences, Section of Human Anatomy, Sapienza University of Rome, Via Caserta 6, 00161 Rome, Italy

**Keywords:** laser, dental implants, thermal damage, peri-implantitis

## Abstract

The aim of this in vitro study was to evaluate thermal effects on implant surfaces using a 445 nm diode laser (Eltech K-Laser Srl, Treviso, Italy) with different power settings and irradiation modalities. Fifteen new implants (Straumann, Basel, Switzerland) were irradiated to evaluate surface alteration. Each implant was divided into two zones: the anterior and posterior areas. The anterior coronal areas were irradiated with a distance of 1 mm between the optical fiber and the implant; the anterior apical ones were irradiated with the fiber in contact with the implant. Instead, the posterior surfaces of all of the implants were not irradiated and used as control surfaces. The protocol comprised two cycles of laser irradiation, lasting 30 s each, with a one-minute pause between them. Different power settings were tested: a 0.5 W pulsed beam (T-on 25 ms; T-off 25 ms), a 2 W continuous beam and a 3 W continuous beam. Lastly, through a scanning electron microscopy (SEM) analysis, dental implants’ surfaces were evaluated to investigate surface alterations. No surface alterations were detected using a 0.5 W laser beam with a pulsed mode at a distance of 1 mm. Using powers of irradiation of 2 W and 3 W with a continuous mode at 1 mm from the implant caused damage on the titanium surfaces. After the irradiation protocol was changed to using the fiber in contact with the implant, the surface alterations increased highly compared to the non-contact irradiation modality. The SEM results suggest that a power of irradiation of 0.5 W with a pulsed laser light emission mode, using an inactivated optical fiber placed 1 mm away from the implant, could be used in the treatment of peri-implantitis, since no implant surface alterations were detected.

## 1. Introduction

In the last few years, the use of dental implants as the main tool to restore masticatory function has significantly grown in contemporary research about materials, shapes and techniques. According to recent estimates, between 12 and 18 million implants are sold each year all around the world, thus showing that implant rehabilitation has become a more and more popular treatment option [1]. Over the years, there has been an evolution of implants’ features that mainly consisted of changes in macro- and microscopic geometries. In fact, to reach better and faster osseointegration, there has been a transition from implants with smooth surfaces to fixtures with rough ones [2,3]. This change in external design, due to surface roughness, also had a negative counterpart, however, since it created new local conditions that facilitate colonization of pathogenic microorganisms, inducing, in many cases, the onset of a severe pathological condition, peri-implantitis: an infectious and inflammatory bacterial disease characterized by progressive local mucosal and bone degeneration that progressively lead to loss of peri-implant supporting tissues and, at the end, to implant loss, with the failure of the restorative project [4,5,6].

Recent studies have underlined that nowadays, about 24% of implant patients suffer from peri-implantitis, and estimates suggest that the problem is continuously growing, with an increase in new diagnoses of between 2.4 and 4 million per year [1,7].

The wide diffusion of this problem is also demonstrated by the great number of studies in the literature focused on the various treatment protocols that have been introduced in daily clinical practice to limit the progression of this pathology and, at the same time, finalized to restore both implants’ stability and life duration. However, despite the different treatment protocols, specifically nonsurgical, surgical, resective, regenerative and combined [8,9,10], the treatment of peri-implantitis still represents a challenge for clinicians, and the therapy success rate is still very low [11]. From a hypothetical point of view, the main target of each peri-implantitis treatment should be removal of the whole bacterial biofilm from the implant surface to achieve decontamination of the implant surface without altering, at the same time, its properties of osteointegration. In fact, it has been largely demonstrated that avoiding change in implants’ surfaces leads to higher treatment success rates if compared to the protocols in which the surface was smoothened [12]. This is probably due to a better process of re-osteointegration of implants in which the original surface shapes are maintained. Among the various peri-implantitis treatment protocols, in the last few years, lasers have been indicated as one of the more effective therapeutic solutions. These devices offer many advantages, such as high patient comfort and compliance, less painful procedures, lower use of sutures and a shorter post-operative course. These advantages obtained with laser applications are probably also due to the capability of the laser beam to reach better the entire implant surface, in comparison to traditional surgical and periodontal instruments, so that even wrinkled and irregular implants’ areas, which would be impossible to treat with traditional mechanical methods, can be easily reached and cleaned, leading to better local decontamination. [13]. In addition, the irradiation of low-intensity diode lasers, combined with the application of specifical substances known as photosensitizers, was also deeply investigated in recent years [14]. This particular treatment protocol is called antimicrobial photodynamic therapy (aPDT) and it is based on the action of a chemical agent, the photosensitizer, in the peri-implant pocket on an implant’s exposed surface; after exposure to a specific laser wavelength, activation of the photosensitizer induces a biochemical reaction with the local oxygen that induces the release of free-radical molecules of singlet oxygen, which are toxic to bacteria. Moreover, a further advantage of aPDT is that the photosensitizer is usually applied topically in a viscous solution, which wets the entire implant surface, thus maximizing the possibility of the therapeutic action reaching all of the irregular areas through the laser beam exposure.

All of the aforementioned protocols have been investigated in several studies in the literature, which has especially focused on the advantages of using different types of lasers in the treatment of peri-implantitis. However, a therapeutic protocol that completely solves the pathology still has not emerged; also in consideration is that that some authors are adverse to laser applications, suggesting the hypothesis of possible damage to implants surfaces induced by the thermal effects determined by laser energies [15,16,17]. In consideration of all these issues, this in vitro study had the aim of testing a new laser wavelength (445 nm) through a scanning electron microscopy (SEM) analysis and evaluating the possible thermal effects, on implant surfaces, produced by different protocols of irradiation.

## 2. Materials and Methods

The laser device used in this study was a K-Laser Blu Dental (Eltech K-Laser, Treviso, Italy), which can operate through the action of laser beams of 3 different wavelengths: 445 (±5) nm in the visible blue, 660 (±5) nm in the visible red and 970 (±5) nm in the Near Infrared (NIR). The three wavelengths can be used in different combinations, but the software also permits selecting one of them. In this study, only the blue wavelength of 445 nm (±5 nm) was used. The diameter of the fiber was 320 µm. The fiber was not activated to avoid reducing the spot of irradiation too much and to concentrate released energy in too-narrow areas, also permitting the irradiation of all of the areas of interest. The laser was used both in contact and in non-contact ways, and the distance of application in the latter, as were the power settings, was modified in the different phases of the test. 

For this study, fifteen new dental implants (Roxolid^®^ line, SLA surface; Straumann, Basel, Switzerland) were implemented. Roxolid is an alloy consisting of 15% zirconium and 85% titanium. A power analysis of 0.858 was calculated with a t-test calculator, where α = 0.2 and with a sample size of 15 implants.

**Figure 1:**
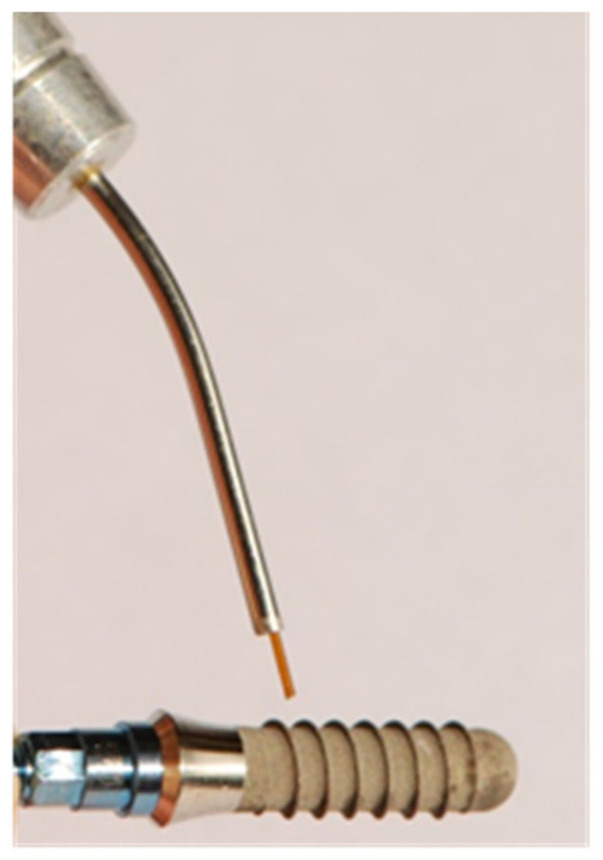
Irradiation of the anterior coronal portion.

**Figure 2:**
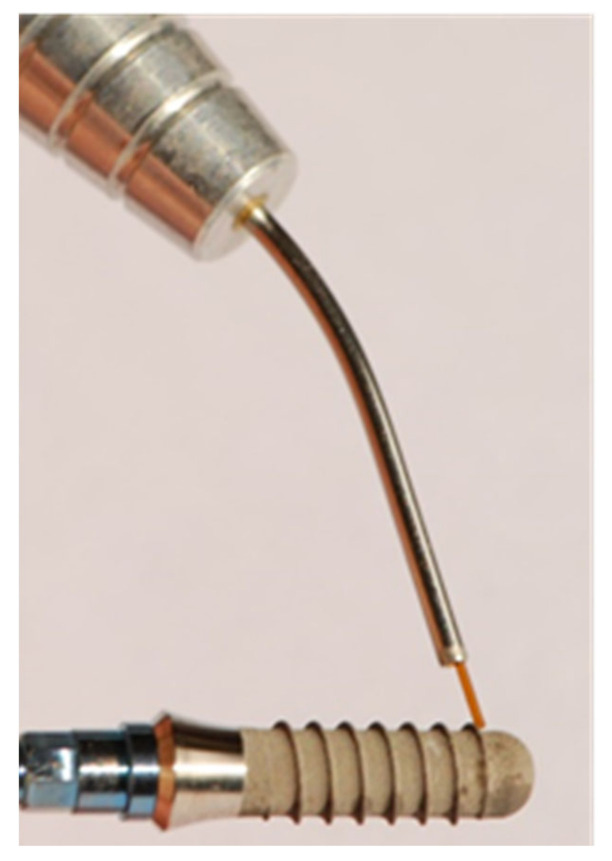
Irradiation of the anterior apical portion.

**Figure 3:**
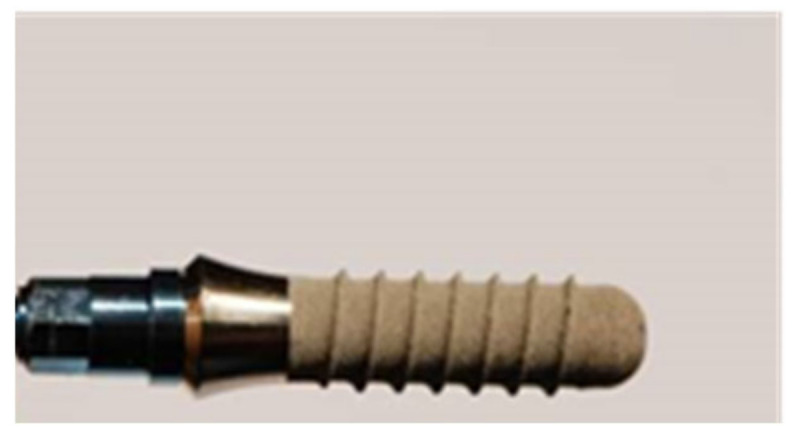
Rear area, control surface.

The SLA surface had a macro-roughness of 20–40 μm peak-to-peak and a micro-roughness of 2–4μm. According to the power of the laser beam used for irradiation, the implants were divided into three groups (groups A, B and C). Each group was composed of five implants. All of the implant surfaces were divided into two macro-areas: the anterior and posterior areas. To distinguish the frontal areas from the back ones, the implant mounter was scratched with a scalpel blade on the front wall only. In addition, the anterior surfaces of the implants were further sub-divided in two areas: one coronal and one apical area. The coronal one included the first three spires of the implant, whereas the apical one included the last three spires of the implant. The anterior coronal areas were irradiated while the handpiece was kept at 1 mm from the implant surface (Figure 1). The anterior apical ones were irradiated with the fiber in contact with the implant (Figure 2). In both cases, the fiber was used at an angle of 45° to the titanium surface. In the literature, most of the treatment protocols for peri-implantitis propose non-contact irradiation, but in clinical practice, it is difficult to always warrant a sure distance of 1 mm between the fiber and the implant due to poor accessibility to the oral cavity and the particular conditions of each peri-implant pocket. Thus, it was decided to also test irradiation in contact in order to closely simulate the operating conditions of daily clinical practice. All of the rear areas were not irradiated but used as control surfaces (CTR) (Figure 3).

The tests were clinical simulations. During the irradiation, the handpiece was moved manually, approximately 5 mm per second, backward and forward. The irradiation protocol was the following: two laser applications of 30 s, with 60 s of pause between them. During the irradiation, no cooling system was adopted for the affected area. The point of the optical fiber was cut before the irradiation of each implant surface. 

To avoid determination of surface alterations during the handling of the samples, they were irradiated directly on the implant holder model contained in their packaging. Group A was irradiated with pulsed waves (PWs), while the other two groups, B and C, were irradiated with continuous waves (CWs) (Table 1). 

The samples of group A, five implants, were irradiated in non-contact mode on the coronal anterior portion with the “peri-implantitis” protocol proposed by K-laser blue Dental: power, 0.5 W; pulsed laser beam (pulsed; T-on, 25 ms; T-off, 25 ms); and movement speed, 5 mm/s.

The group B samples, five implants, were irradiated in non-contact mode on the anterior coronal wall and in contact mode in the anterior apical area, with the following parameters: power, 2 W; continuous laser beam; movement speed, 5 mm/s. 

As for the second group, the five implants in group C were irradiated in non-contact mode on the anterior coronal wall and in contact mode on the anterior apical wall, with the following parameters: power, 3 W; continuous wave; movement speed, 5 mm/s. The irradiation protocols tested on the second and third group were created specifically for this study; in fact, during clinical practice, it is not possible to use such high energies without causing damage to the soft tissues that surround the implant. However, the settings of 2.0 W and 3.0 W, like the continuous emission modality, were expressly chosen to stress the laser thermal effect and to obtain more evident results.

After the irradiation, the implants were cleaned in an ultrasonic bath with isopropanol for 5 min to remove dirt and fats from their surfaces and to avoid contamination during SEM observations. Each sample was mounted with carbon tape on an aluminum stub and was observed under a Hitachi SU 4000 Field Emission Scanning Electron Microscope and under a Hitachi SU3500 scanning electron microscope. The operating conditions were high-vacuum and an acceleration voltage of 5–20 kV. The exact site of laser irradiation was observed, and pictures were taken at different magnifications ranging from 25× (500 µm scale bar) to 5000× (3 µm scale bar). 

## 3. Results

**Figure 4:**
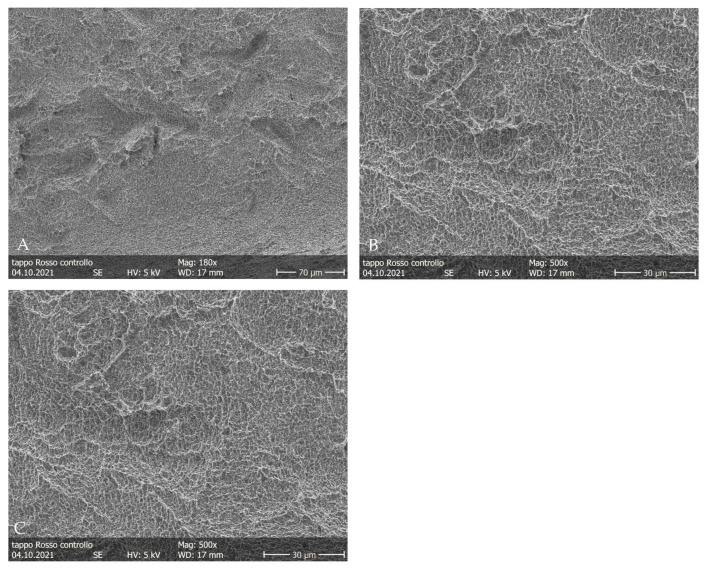
(**A**–**C**) SEM images of control implant samples. Magnifications from 180× to 4000×.

**Figure 5:**
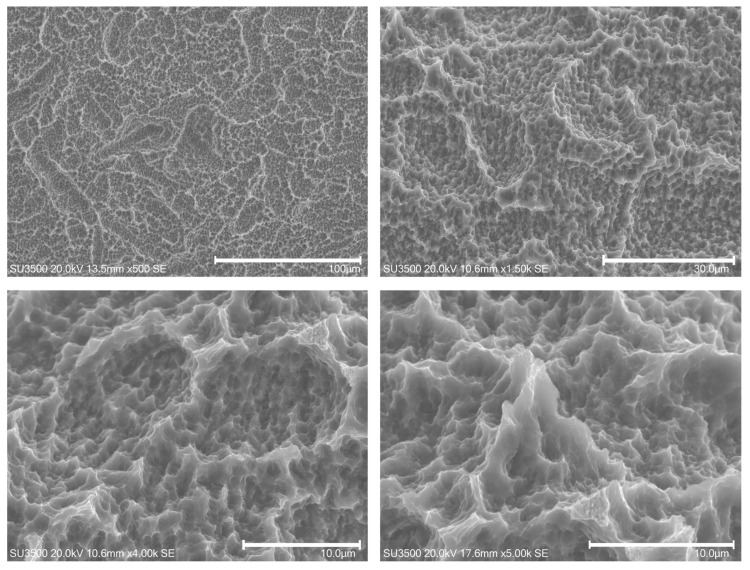
Group A. Surface patterns of sandblasted titanium implants after blue laser irradiation at a distance, showing no differences in the surface patterns compared to the non-lasered controls. 0.5 W power setting. Magnifications from 500× to 5000×.

The untreated control implant surfaces (CTR) revealed the typical microporous structures of SLA surfaces obtained through a combined sandblasting and etching process (Figure 4). The surfaces’ morphologies of these samples were irregular, with sharp edges and ridges. The areas irradiated with 0.5 W in non-contact mode did not show substantial differences compared to the posterior area (Figure 5) (CTR). At the end of the irradiation, the optical fiber still retained its macroscopic initial characteristics.

**Figure 6:**
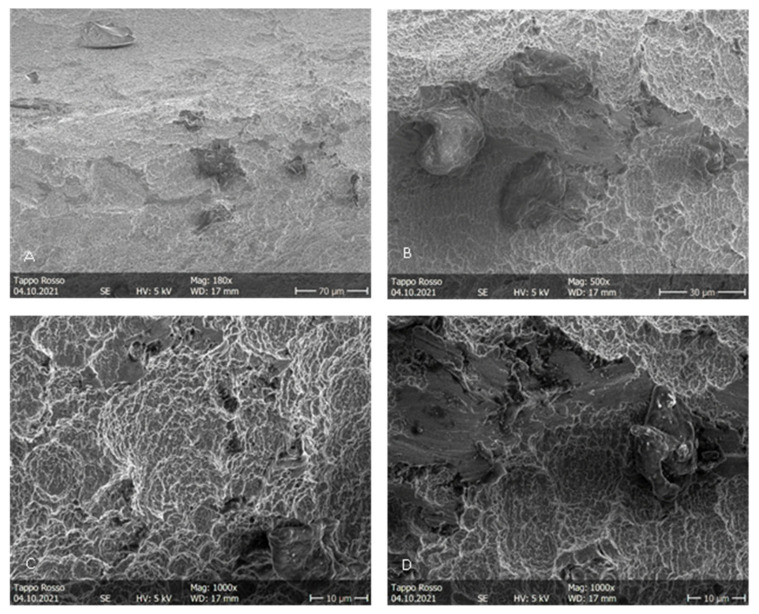
Group B. (**A**–**D**) Surface patterns of sandblasted titanium implants after blue laser irradiation at a distance, showing thermal signs, loss of porosity and relatively smooth surfaces. 2.0 W power setting. Magnifications from 180× to 1000×.

**Figure 7:**
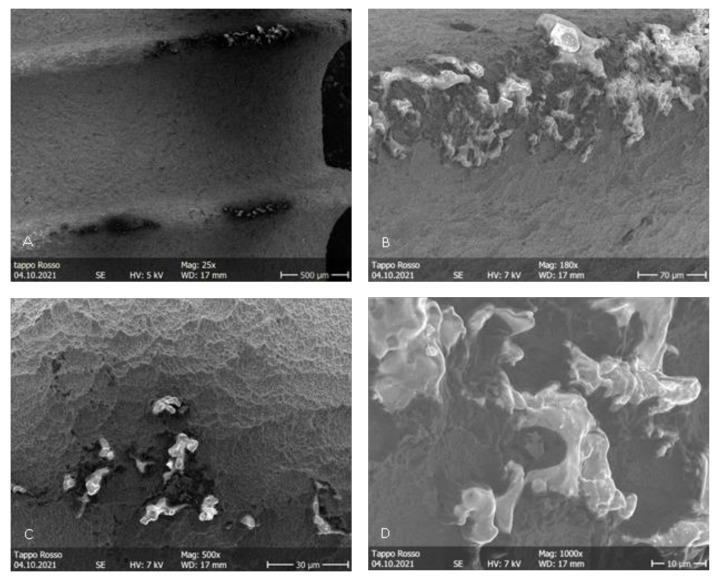
Group B. (**A**–**D**) Surface patterns of sandblasted titanium implants after blue laser irradiation in contact, showing thermal signs and damage to the sandblasted surfaces. 2.0 W power setting. Magnifications from 25× to 1000×.

**Figure 8:**
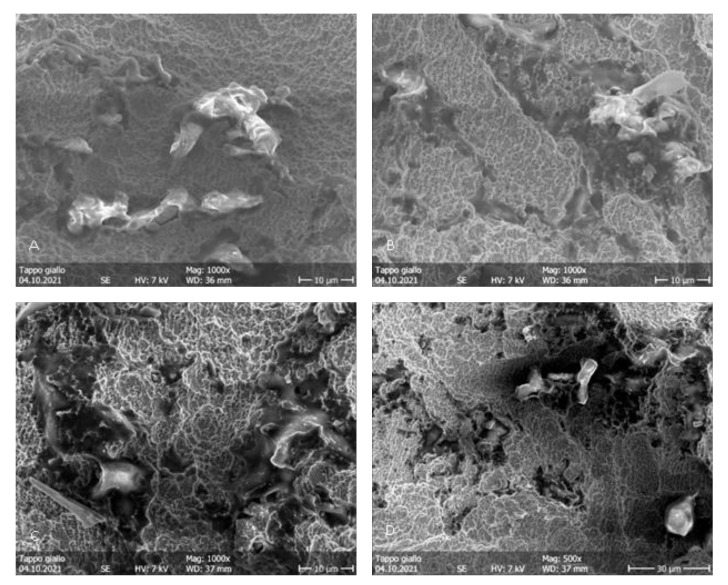
Group C. (**A**–**D**) Surface patterns of sandblasted titanium implants after blue laser irradiation at a distance, showing thermal signs, loss of porosity and relatively smooth surfaces. 3.0 W power setting. Magnifications from 500× to 1000×.

**Figure 9:**
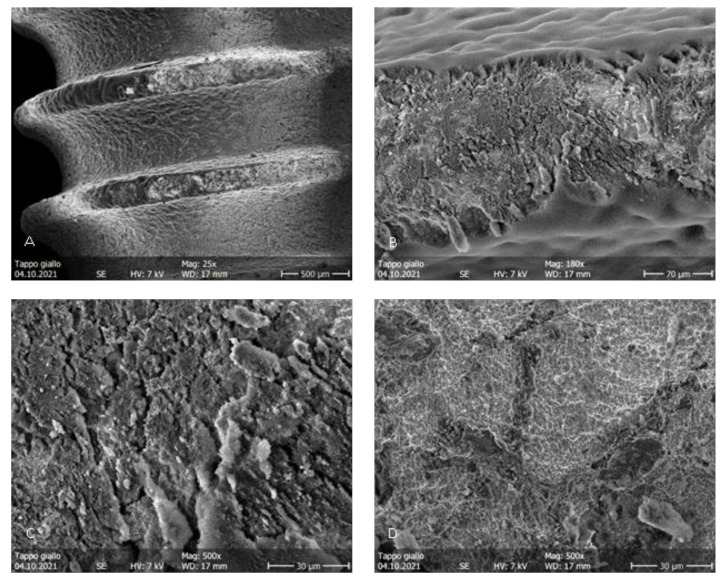
Group C. (**A**–**D**) Surface patterns of sandblasted titanium implants after blue laser irradiation in contact, showing thermal signs, microfractures and crater-like defects, which represent the extensive damage to the surfaces. 3.0 W power setting. Magnifications from 25× to 500×.

During the irradiation of the implants belonging to groups B and C, the fiber was activated autonomously, probably due to the high power used and the dark color of the irradiated surface. This concentrated all of the energy in a smaller area than in the starting conditions, determining severe thermal effects immediately visible to the naked eye. More precisely, in Figure 6A, it is possible to appreciate the probable detachment of a titanium surface fragment. In Figure 6B,D, it is possible to appreciate the loss of surface roughness due to the thermal signs of the titanium ridges. In the images of the implants in group B, irradiated in contact (Figure 7), the alterations are even more pronounced. At 25×, it was already possible to detect a loss of roughness due to the thermal signs of the titanium ridges and the carbonization of the optical fiber, probably caused by the high temperatures reached by the irradiated surfaces. In the images of the implants of the group C (Figure 8), the thermal effects of the action of the laser beam are even more evident. Figure 9B shows the coil of an implant of group C at a magnification of 180× and in comparison with a control surface; it is possible to observe the important loss of surface roughness and, in addition, microfractures and crater-like defects, which represent extensive damage to the surfaces. While in the second group, there were point-like alterations of the surface, in the third, it was possible to appreciate more extensive alterations that probably mimicked the path taken by the laser beam. In addition to the defects shown in the microscopic observation, the implants in group C at the end of the irradiation process showed macroscopically detectable chromatic alterations. A schematization of the damaged areas is available on Table 2 (Table 2).

## 4. Discussion

The health of peri-implant tissues plays a key role in keeping implants on site for a long time.

Peri-mucositis and peri-implantitis are both plaque-associated inflammatory conditions that involve the tissues around implants. Their definition was well-shown by workgroup 4 of the World Workshop on the Classification of Periodontal and Peri-Implant Diseases and Conditions in 2017 [4]. The consensus summarized the scientific knowledge about conditions that could affect tissues around implants. For the first time in the scientific literature, it also defined the parameters to consider an implant in a “health status”.

It is well-known that inflammation is the biological response to an infective stimulus. Accumulation of plaque and calculus leads to inflammation of the supporting tissues; this process is called mucositis, which, if not resolved through conventional daily and professional hygiene, can evolve into peri-implantitis, resulting in bone resorption, and, in the worst scenario, it may lead to the loss of the implant.

The peri-implant inflammation process is usually faster and more destructive compared to the periodontal one due to the absence of periodontal ligaments [18]. The peri-implantitis treatment targets are mainly removal of bacterial biofilm and resolution of the inflammatory process. This is the sole way that may lead to the closure of the pocket, resolution of bleeding and of swelling and stopping progressive bone resorption, thus increasing the survival rate of the implant [19]. Unfortunately, even if the aims of therapeutic protocols are clear and well-established, the treatment of peri-implant diseases still represents one of the hardest challenges for dental clinicians and researchers.

Laser devices have been proposed in the last fifty years in different dental applications. Laser light, thanks to its peculiar properties, has shown important advantages in restorative dentistry, endodontics, oral surgery and pathology, periodontology and implantology [20,21].

The use of lasers of different wavelengths as tools to increase the success rate of peri-implantitis treatment procedures has been deeply investigated. Many studies have underlined lasers’ anti-infective proprieties, especially against periodontal pathogens, alone or in combination with other techniques (mechanical and ultrasonic debridement, air polishing) [22,23]. The most common wavelengths that were proposed over the years for this kind of treatment were diodes ranging from 810 nm to 950 nm; the New:YAG in the Near Infrared (NIR); and the Erbium group, both the Er:YAG and the Er:YSGG, in the Mid-Infrared, at 2940 and 2790 nm, respectively. In a split-mouth clinical trial on 20 patients affected by periodontitis, Moreira et al. demonstrated the efficacy of an adjunctive antimicrobial photodynamic therapy toward a non-surgical treatment. They found a reduction in the bacteria of the red and orange complexes in the sites treated with the adjunct of a low-intensity diode laser compared to the ones treated only with conventional therapy of scaling and root planning [24].

Moreover, laser application with a low energy level can exert biological effects through biostimulation of the soft tissue around implants. This process, known also as photobiomodulation, may lead to a faster wound-healing process, as was demonstrated in many studies [25,26,27,28].

In recent years, one of the most interesting novelties in laser dentistry has been the introduction of blue visible laser light, ranging from 400 to 450 nm, both in surgery and in periodontology. From a physical point of view, blue light action is more superficial, being highly scattered by water, and it is also well-absorbed by hemoglobin, which has one of its peaks of absorption at around 450 nm. This characteristic has made the blue laser particularly suitable for surgical procedures for high coagulative action. Moreover, the blue light has been demonstrated to have also specific antibacterial effects, so many studies have hypothesized its application in periodontology to reduce bacterial plaque in the periodontal pockets. Among the various potentialities of this versatile wavelength, it is also of particular interest that biomodulating intracellular action, through the activation of specific membrane receptors, in particular opsins, leads to transcriptions of nuclear genes, both by direct action and through the intermediate actions of intracellular mediators such as ROSs and Nitric Oxide (NO), that lead to beneficial effects for irradiated tissues.

Sang Woong Park et al. demonstrated the potentiality of a 445 nm blue visible laser to improve local blood flow through a vasorelaxation process when used in a pulsed mode with a low level of energy [29].

Even though the 445 nm laser is a relatively new device, several authors have tested this wavelength on the titanium surfaces of implants to investigate the long-lasting controversy concerning possible surface alterations secondary to the thermal effects induced by laser applications. Malmqvist et al. [30] found no surface alterations when irradiating titanium discs for 4 min at 2.0 W and at a distance of 1 mm in a non-contact way. The same results were obtained by Deppe et al. [31] by radiating in either continuous-wave (CW) mode (0.8 W, 5 s) or pulsed mode (3 W, 20 s, 10% duty cycle and 1 W, 10 s, 50% duty cycle). In their in vitro study, Deppe and co-workers also evaluated the temperature increase of the implant surface when it was irradiated with a blue laser [31], In fact, it is important to remember that an increase in bone temperature of even 10° for 60 s may induce severe, permanent structure alterations, greatly compromising the survival of implants in the bone. In any case, in this study, the authors observed that an exposure of 5 s in CW mode at 0.8 W was safe for implant surfaces. Similar results were also obtained when the laser was applied in pulsed waves at 3 W for 20 s with a 10% duty cycle and at 1 W for 10 s with a 50% duty cycle. [32].

In contrast with the results registered in these studies, in the present experience, evident surface alterations to dental implants have been reported at both 2.0 W and 3.0 W power used in CW mode. The actions of 2 W and 3 W laser beams determined the decrease in the roughness of the implant surface due to the thermal signs of the titanium ridges. Higher power settings were associated with more evident surface damage, even macroscopically visibly. In any case, in this study, the contact-mode irradiation results were always associated with more evident alterations, independently from the adopted settings. At this moment, no clinical trial has evaluated the ability of re-osteointegration of implants with altered surfaces. Regardless of this, the implants’ macrostructural and microstructural changes will probably interfere with the processes of re-osteointegration of the implants in the case of resolution of the main pathology.

In our study, only when the implants’ surfaces were radiated for two cycles of 30 s at 0.5 W in a non-contact way, at 1 mm from the implant, were no surface alterations observed. According to the obtained results, the K-laser developer recommended 0.5 W as the standard power setting for the treatment of peri-implantitis. Furthermore, among the power settings chosen in this in vitro study, 0.5 W represented the suitable setting that could be used in a general clinic scenario.

Another problem encountered with the use of the blue laser at higher powers was the carbonization of fiber on the implant surface. This effect causes self-activation of the fiber info the pocket, leading to a high local temperature increase, which could represent an inflammatory stimulus for peri-implant tissues and cause increased accumulation of plaque.

Surface alterations of titanium discs and dental implants were found in many other studies in the literature, which used different laser parameters. Romanos et al. [33] reported that the application of Nd: YAG (1064 nm wavelength) at 2.0 W and 4.0 W in contact mode determined thermal signs and damage to the surfaces of titanium sperimental discs. Conversely, they found no surface alterations when using a diode laser (980 nm) with 5.0, 10.0 and 15.0 W in continuous mode with contact radiation. Lee et al. [34] reported surface alterations with Er: YAG (2940 nm) at 1.4 W and 1.8 W (140 mJ/pulse and 180 mJ/pulse) in contact for 1 min, but not with 1.0 W. The same results were obtained by Stubinger et al. [35,36,37] with Er: YAG at 3.0 W and 5.0 W (300 mJ/pulse and 500 mJ/pulse), used for 10 s in contact with the disc. They found no surface alterations with Er: YAG at 1.0 W, as well as when using a CO_2_ laser (10,600 nm) at 2.0, 4.0 or 6.0 W or a diode laser (810 nm) at 1.0 or 3.0 W; both the CO_2_ and diode lasers were used at a distance.

In this in vitro study, the temperature changes of the implants during the irradiation process were not evaluated, thus representing a key limitation of this research.

More studies are required to understand the real capability of lasers to assist clinicians in the treatment of peri-implantitis. It is also mandatory to compare different laser wavelengths, power settings and radiating modalities with the same clinical settings to better understand the real efficiencies of various laser devices and the ideal protocols of application of these devices for the maximum exploitation of their potentialities in this challenging problem, permitting the widest application of this technique, with great advantages for both clinicians and patients.

## 5. Conclusions

According to the results obtained through the magnified images, it was possible to establish that for implants irradiated with 2.0 W and 3.0 W, with the fiber both at a distance from and in contact with the implant, surface alterations, loss of porosity, microfractures and crater-like defects were determined, representing severe damage to the surface. The power of irradiation of 0.5 W with a pulsed laser light emission mode (T-on, 25 ms–T-off, 25 ms), using an inactivated optical fiber placed at 1 mm from the implant, produced no surface alterations. This could probably be considered a safety setting in order to not alter the implant surface and to continue studies the therapy for peri-implantitis. Lastly, contact irradiation and higher power cause more evident alterations than do distant irradiation and lower power. Of course, microbiological tests are required to state the efficacy of the germs that induce and support the peri-implantitis pathological process.

## Figures and Tables

**Table 1:** Schematization of the irradiated areas.

	0.5 W	2.0 W	3.0 W
*In-Contact*	0	5	5
*Non-Contact*	5	5	5
*Control*	5	5	5
*Total*	10	15	15

**Table 2:** Schematization of the damaged areas.

	0.5 W	2.0 W	3.0 W
*In-Contact*	0	5	5
*Non-Contact*	0	5	5
*Control*	0	5	5
*Total*	0	15	15

## Data Availability

The data shown are at the complete disposal of the scientific community and can be found through the corresponding author.
